# An Empirical Investigation Into Deep and Shallow Rule Learning

**DOI:** 10.3389/frai.2021.689398

**Published:** 2021-10-22

**Authors:** Florian Beck, Johannes Fürnkranz

**Affiliations:** Institute for Application-oriented Knowledge Processing (FAW), Johannes Kepler University, Linz, Austria

**Keywords:** inductive rule learning, deep learning, learning in logic, mini-batch learning, stochastic optimization

## Abstract

Inductive rule learning is arguably among the most traditional paradigms in machine learning. Although we have seen considerable progress over the years in learning rule-based theories, all state-of-the-art learners still learn descriptions that directly relate the input features to the target concept. In the simplest case, concept learning, this is a disjunctive normal form (DNF) description of the positive class. While it is clear that this is sufficient from a logical point of view because every logical expression can be reduced to an equivalent DNF expression, it could nevertheless be the case that more structured representations, which form deep theories by forming intermediate concepts, could be easier to learn, in very much the same way as deep neural networks are able to outperform shallow networks, even though the latter are also universal function approximators. However, there are several non-trivial obstacles that need to be overcome before a sufficiently powerful deep rule learning algorithm could be developed and be compared to the state-of-the-art in inductive rule learning. In this paper, we therefore take a different approach: we empirically compare deep and shallow rule sets that have been optimized with a uniform general mini-batch based optimization algorithm. In our experiments on both artificial and real-world benchmark data, deep rule networks outperformed their shallow counterparts, which we take as an indication that it is worth-while to devote more efforts to learning deep rule structures from data.

## 1 Introduction

Dating back to the AQ algorithm (Michalski, 1969), inductive rule learning is one of the most traditional fields in machine learning. However, when reflecting upon its long history (Fürnkranz et al., 2012), it can be argued that while modern methods are somewhat more scalable than traditional rule learning algorithms (see, e.g., Wang et al., 2017; Lakkaraju et al., 2016), no major break-through has been made. In fact, the RIPPER rule learning algorithm (Cohen, 1995) is still very hard to beat in terms of both accuracy and simplicity of the learned rule sets. All these algorithms, traditional or modern, typically provide flat lists or sets of rules, which directly relate the input variables to the desired output. In concept learning, where the goal is to learn a set of rules that collectively describe the target concept, the learned set of rules can be considered as a logical expression in disjunctive normal form (DNF), in which each conjunction forms a rule that predicts the positive class.

In this paper, we argue that one of the key factors for the strength of deep learning algorithms is that latent variables are formed during the learning process. However, while neural networks excel in implementing this ability in their hidden layers, which can be effectively trained via backpropagation, there is essentially no counter-part to this ability in inductive rule learning. We therefore set out to verify the hypothesis that deep rule structures might be easier to learn than flat rule sets, in very much the same way as deep neural networks have a better performance than single-layer networks (Mhaskar et al., 2017). Note that this is not obvious, because, in principle, every logical formula can be represented with a DNF expression, which corresponds to a flat rule set, in the same way as, in principle, one (sufficiently large) hidden layer is sufficient to approximate any function with a neural network (Hornik, 1991). As no direct comparison is possible because of the lack of a powerful algorithm for learning deep rule sets, our tool of choice is a simple stochastic optimization algorithm to optimize a rule network of a given size. While this does not quite reach state-of-the-art performance (in either setting, shallow or deep), it nevertheless allows us to gain some insights into these settings. In particular, we aim to see whether deep structures can be better learned than shallow structure in an identical setting using the same general optimization algorithm. To that end, we test deep and shallow rule networks on both, real-world UCI benchmark datasets, as well as artificial datasets for which we know the underlying target concept representations. Moreover, we also briefly look at the interpretability of the learned concepts in both their learned structure as well as their equivalent DNF formulation, but find that the presentation of logical formulas in a human interpretable way is still largely an open question.

The remainder of the paper is organized as follows: *Deep Rule Learning* elaborates why deep rule learning is of particular interest and refers to related work. We propose a new network approach in *Deep Rule Networks* and test it in *Experiments*. The results are concluded in *Conclusion*, followed by possible future extensions and improvements in *Future Work*.

## 2 Deep Rule Learning

In this section, we will briefly discuss the state-of-the-art in learning deep, structured rule bases. We start with a brief motivation, and continue to review related work in several relevant areas, including constructive induction, multi-label rule learning, or binary and ternary networks.

### 2.1 Motivation

**FIGURE 1:**
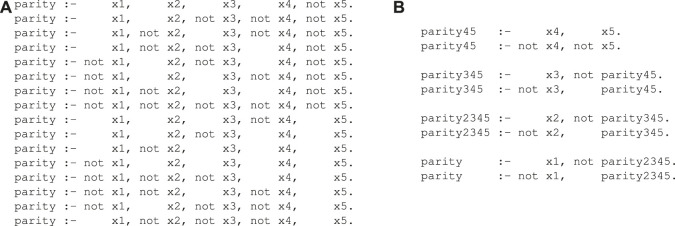
Unstructured and structured rule sets for the parity concept. **(A)** A flat unstructured rule set for the parity concept. **(B)** A deep unstructured rule base for parity using three auxiliary predicates.

Rule learning algorithms typically provide flat lists that directly relate the input to the output. Consider, e.g., the following example: the parity concept, which is known to be hard to learn for heuristic, greedy learning algorithms, checks whether an odd or an even number of *R* relevant attributes (out of a possibly higher total number of attributes) are set to true. Figure 1A shows a flat rule-based representation^1^ of the target concept for *R* = 5, which requires 2^*R*−1^ = 16 rules. On the other hand, a structured representation, which introduces three auxiliary predicates (parity2345, parity345 and parity45 as shown in Figure 1B), is much more concise using only 2 ⋅ (*R* − 1) = 8 rules. We argue that the parsimonious structure of the latter could be easier to learn because it uses only a linear number of rules, and slowly builds up the complex target concept parity from the smaller subconcepts parity2345, parity345 and parity45.

**FIGURE 2:**
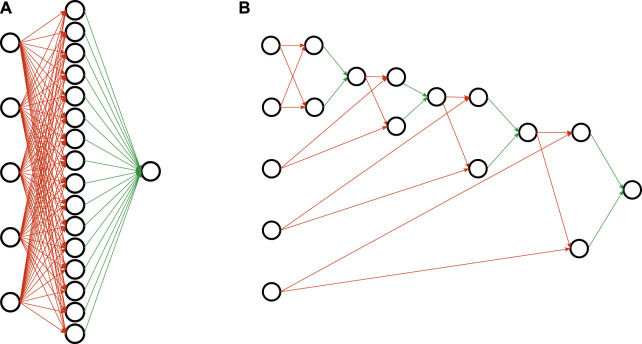
Network representations of the parity rule sets of Figure 1. Red connections are logical ANDs, green edges correspond to logical ORs. **(A)** Shallow representation. **(B)** Deep representation.

To motivate this, we draw an analogy to neural network learning, and view rule sets as networks. Conventional rule learning algorithms learn a flat rule set of the type shown in Figure 1A, which may be viewed as a concept description in disjunctive normal form (DNF): Each rule body corresponds to a single conjunct, and these conjuncts are connected via a disjunction (each positive example must be covered by one or more of these rule bodies). This situation is illustrated in Figure 2A, where the five input nodes are connected to 16 hidden nodes - one for each of the 16 rules that define the concept - and these are then connected to a single output node. Analogously, the deep parity rule set of Figure 1B may be encoded into a deeper network structure as shown in Figure 2B. Clearly, the deep network is more compact and considerably sparser in the number of edges. Of course, we need to take into consideration that the optimal structure is not known beforehand and presumably needs to emerge from a fixed network structure that offers the possibility for some redundancy, but nevertheless we expect that such structured representations offer similar advantages as deep neural networks offer over single-layer networks.

It is important to note that deep structures do not increase the expressiveness of the learned concepts. Any formula in propositional logic (and we limit ourselves to propositional logic in this project) can be converted to a DNF formula. In the worst case (a so-called *full DNF*), each of the input variables appears exactly once in all of the inputs, which essentially corresponds to enumerating all the positive examples. Thus, the size of the number of conjuncts in a DNF encoding of the inputs may grow exponentially with the number of input features. This is in many ways analogous to the universal approximation theorem (Hornik, 1991), which essentially states that any continuous function can be approximated arbitrarily closely with a shallow neural network with a single hidden layer, provided that the size of this layer is not bounded. So, in principle, deep neural networks are not necessary, and indeed, much of the neural network research in the 90s has concentrated on learning such two-layer networks. Nevertheless, we have now seen that deep neural networks are easier to train and often yield better performance, presumably because they require exponentially less parameters than shallow networks (Mhaskar et al., 2017). In the same way, we expect that deep logical structures will yield more efficient representations of the captured knowledge and might be easier to learn than flat DNF rule sets.

### 2.2 State-Of-The-Art in Deep Rule Learning

As mentioned above, the problem of deep rule learning has only rarely been explicitly addressed in the literature. Modern rule learning algorithms rely on ensemble-based sequential loss minimization. Friedman and Popescu (2008), e.g., learn a sparse linear model from features that have been obtained from the rules corresponding to the leaves of a decision tree ensemble such as a random forest (Breiman, 2001). Algorithms like ENDER (Dembczyński et al., 2010) or BOOMER (Rapp et al., 2020) integrate the rule induction into the boosting procedure by aiming at the minimization of an overall regularized loss function during the learning of individual rules. The learning algorithm for finding interpretable decision sets (Lakkaraju et al., 2016) explicitly includes several biases for interpretability into the objective function and proposes smooth stochastic search, a method for efficiently finding an approximative solution. Angelino et al. (2017) demonstrate an algorithm that is able to find exact loss minimizing rules.

All these algorithms are single-concept learners, i.e., they learn rules for a single target concept. However, as has been argued by Fürnkranz et al. (2020), works in several related areas are quite relevant to the problem. In the following, we briefly review approaches that are able to convert deep models into rules (*Rule Extraction From Deep Models*), to autonomously discover hidden, auxiliary concepts (*Learning Intermediate Concepts*), or to learn multiple dependent target concepts (*Learning Multiple Dependent Concepts*), and even review a few algorithms that learn logical networks (*Discrete Deep Networks*).

#### 2.2.1 Rule Extraction From Deep Models

The strength of many recent learning algorithms, most notably deep learning (Lecun et al., 2015; Schmidhuber, 2015), but also kernel-based methods (Cristianini and Shawe-Taylor, 2000) is that the input variables are combined to form latent concepts during the learning process. Understanding the meaning of these hidden variables is crucial for transparent and justifiable decisions. Consequently, some research has been devoted to trying to convert arcane models such as neural networks (Andrews et al., 1995; Craven and Shavlik, 1997; Schmitz et al., 1999) or support-vector machines (Barakat and Bradley, 2010; Guerreiro and Trigueiros, 2010) to more interpretable rule-based or tree-based models. Nevertheless, these models typically treat the input models as black-boxes, and do not try to uncover the structure in the hidden layers. One exception is, e.g., DeepRED (Zilke et al., 2016 ; González et al., 2017), which tries to learn a decision tree model for each node in a neural network, but eventually compiles them into a single flat rule set. In a particularly interesting recent work, Polato and Aiolli (2019) defined kernels based on propositional logic, which allowed them to demonstrate several examples of the hardness of explicitly formulating some of the learned concepts (such as “three cards of a kind” in poker) in pure Boolean logic.

Instead of making the entire model interpretable, methods like LIME (Ribeiro et al., 2016) are able to provide local explanations for inscrutable models, allowing to trade off fidelity to the original model with interpretability and complexity of the local model. For example, LORE (Guidotti et al., 2018) explicitly targets rule-based explanations of black-box models. An interesting aspect of rule-based theories is that they can be considered as hybrids between local and global explanations (Fürnkranz, 2005): A rule set may be viewed as a global model, whereas the individual rule that fires for a particular example may be viewed as a local explanation. A recent method, GLocalX (Setzu et al., 2021), conversely, aims to combine local explanations into a global rule model. However, again, these approaches are not able to capture the subconcepts detected by the black-box classifiers.

#### 2.2.2 Learning Intermediate Concepts

For learning structured rule sets, a key challenge is how to define and train the intermediate, hidden concepts *h*_*i*_ which may be used for improving the final prediction. Note that in a conventional, flat structure as in Figure 2A, all *h*_*i*_ always had a fairly clear semantic in that they capture some aspect of the target variable *y*. The rule that predicts *h*_*i*_ essentially defines a local pattern for *y* (Fürnkranz, 2005).

However, when learning deeper structures, other hidden concepts need to be defined which do not directly correspond to the target variable, as can be seen from the structured parity concept of Figure 1B. This line of work has been known as *constructive induction* (Matheus, 1989) or *predicate invention* (Stahl, 1996), but surprisingly, it has not received much attention since the classical works in inductive logic programming in the 1980s and 1990s. One approach is to use a wrapper to scan for regularly co-occurring patterns in rules, and use them to define new intermediate concepts which allow to compress the original theory (Pfahringer, 1994; Wnek and Michalski, 1994). Alternatively, one can directly invoke so-called predicate invention operators during the learning process, as, e.g., in Duce (Muggleton, 1987), which operates in propositional logic, and its successor systems in first-order logic (Muggleton and Buntine, 1988; Kijsirikul et al., 1992; Kok and Domingos, 2007). Similar to Duce, systems like MOBAL (Morik et al., 1993) not only try to learn theories from data, but also provide functionalities for reformulating and restructuring the knowledge base (Sommer, 1996). More recently, Muggleton et al. (2015) introduce a technique that employs user-provided meta rules for proposing new predicates, which allow it to invent useful predicates from only very training examples. Kramer (2020) provides an excellent recent summary of work in this area.

#### 2.2.3 Learning Multiple Dependent Concepts

Much of the work in machine learning is devoted to single prediction tasks, i.e., to tasks where an input vector is mapped to a single output value. When aiming to learn a deep rule base, however, one has to tackle the problem of learning a network of multiple, possibly mutually dependent concepts. A pioneering work in this area is Malerba et al. (1997), which gives a broad discussion of the problem of learning multiple dependent concepts in the form of a dependency graph. Back then, the problem has primarily been studied in inductive logic programming and relational learning (see, e.g., De Raedt et al., 1993), but it has recently reappeared in multilabel classification (Tsoumakas and Katakis, 2007; Tsoumakas et al., 2010; Zhang and Zhou, 2014) and, more generally, in multi-target prediction (Waegeman et al., 2019).

In fact, most of the research in multi-label classification aims for the development of methods that are capable of modeling label dependencies (Dembczyński et al., 2012). One of the best-known approaches are so-called classifier chains (CC) (Read et al., 2011, 2021), which learn the labels in some (arbitrary) order where the predictions for previous labels are included as features for subsequent models. Several extensions of this framework have been studied, such as Burkhardt and Kramer (2015), who propose to cluster labels into sequential blocks of sets of labels. A general framework proposed by Read and Hollmén (2014, 2015) formulates multi-label classification problems as deep networks where label nodes are a special type of hidden nodes which can appear in multiple layers of the networks.

However, while these algorithms all aim at learning multiple interconnected models, they are not capable of explicitly defining intermediate, auxiliary concepts. Some works that aim at finding so-called label embeddings (e.g., Nam et al., 2016) may be viewed in this context, but they do not learn rule-based descriptions. Rules are particularly interesting for solving this kind of problems because they allow to explicitly formalize and model dependencies between labels and between data and labels in an explicit and seamless way (Hüllermeier et al., 2020). Rapp et al. (2020) propose an efficient boosting-based rule learner for multi-label classification.

#### 2.2.4 Discrete Deep Networks

Finally, in the wake of the success of deep neural networks, a few approaches have been developed that explicitly aim at learning networks with a logical structure. Sum-product networks (SPNs; Poon and Domingos, 2011) have an analogous structure to our AND/OR networks, but aim at modeling probability distributions instead of logical expressions. Of particular interest to our study is the work of Delalleau and Bengio (2011), who compare deep and shallow SPNs, and find that deep structures can result in more compact representations, which is in line with the motivation of our work.

Somewhat closer to logic are frameworks such as TensorLog (Cohen et al., 2020) that aim at making probabilistic logical reasoning differentiable and therefore amenable to implementation and optimization in a deep learning environment. For example, the approach of Evans and Grefenstette (2018) is able to learn logical theories from data in a matter that is considerably more robust than traditional techniques from inductive logic programming. However, it only learns to weight rules that can be generated from a set of predefined templates. In particular, no auxiliary, hidden predicates can emerge from the learner. Fuzzy pattern trees (Senge and Hüllermeier, 2011) may be viewed in this way in that they build up a hierarchical structure of generalized logical functions, so that their internal nodes may be viewed as intermediate fuzzy logical concepts.

Most relevant to our work are binary networks (Courbariaux et al., 2015; Qin et al., 2020), which restrict the weights to values {−1, 1}. However, their semantics does typically not correspond to conventional logic rules, in that they enforce every feature to contribute to the function to be learned, either in its positive or negated form. Ternary networks (Li et al., 2016; Zhu et al., 2017), with weights {−1, 0, 1}, where 0 corresponds to ignoring the corresponding feature in the rule, could provide a solution to this, and are, indeed, quite similar in spirit to the networks we train in the remainder of this paper. Typically, they train a full deep neural network, and subsequently quantize the resulting weights to the desired two or three values, in order to allow a more compact representation and faster inference. Nevertheless, we have not made use of them in our work, because we wanted to focus on a simple optimization algorithm that is invariant for deep and shallow structures. For essentially the same reason, we have also not used state-of-the-art flat rule learning algorithms, so that observed differences in performance can be clearly attributed to differences in the network structure, and not in the optimization algorithms.

## 3 Deep Rule Networks

For our studies of deep and shallow rule learning, we define rule-based theories in a networked structure, which we describe in the following. We build upon the shallow two-level networks which we have previously used for experimenting with mini-batch rule learning (Beck and Fürnkranz, 2020), but generalize them from a shallow DNF-structure to deeper networks.

### 3.1 Network Structure

A conventional rule set consisting of multiple conjunctive rules that define a single target concept, corresponds to a logical expression in disjunctive normal form (DNF). An equivalent network consists of three layers, the input layer, one hidden layer (= AND layer) and the output layer (= OR layer), as, e.g., illustrated in Figure 2A. The input layer receives one-hot-encoded nominal attribute-value pairs as binary features (= literals), the hidden layer conjuncts these literals to rules and the output layer disjuncts the rules to a rule set. The network is designed for binary classification problems and produces a single prediction output that is true if and only if an input sample is covered by any of the rules in the rule set.

For generalizing this structure to deeper networks, we need to define multiple layers. While the input layer and the output layer remain the same, the number and the size of the hidden layers can be chosen arbitrarily. In the more general case, the hidden layers are treated alternately as conjunctive and disjunctive layers. We focus on networks with an odd number of hidden layers, starting with a conjunctive hidden layer and ending with a disjunctive output layer. In this way, the output will be easier to compare with rule sets in DNF. Furthermore, the closer we are to the output layer, the more extensive are the rules and rule sets, and the smaller is the chance to form new combinations from them that are neither tautological nor contradictory. As a consequence, the number of nodes per hidden layer should be lower the closer it is to the output layer. This makes the network shaped like a funnel.

### 3.2 Network Weights and Initialization

In the following, we assume the network to have *n* + 2 layers, with each layer *i* containing *s*_*i*_ nodes. Layer 0 corresponds to the input layer with *s*_0_ = |x| and layer *n* + 1 to the output layer with *s*_*n*+1_ = 1. Furthermore, a weight $w_{jk}^{\left(i\right)}$ is identified by the layer *i* it belongs to, the node *j* from which it receives the output, and the node *k* in the successive layer *i* + 1 to which it passes the activation. Thus, the weights of each layer can be represented by an *s*_*i*_ × *s*_*i*−1_-dimensional matrix $W^{\left(i\right)}=\left[w_{jk}^{\left(i\right)}\right]$ . In total, there are $\sum_{i=0}^{n}s_{i}s_{i+1}$ Boolean weights which have to be learned, i.e., have to be set to true (resp. 1) or false (resp. 0). If weight $w_{jk}^{\left(i\right)}$ is set to true, this means that the output of node *j* is used in the conjunction (if *i* mod 2 = 0) or disjunction (if *i* mod 2 = 1) that defines node *k*. If it is set to false, this output is ignored by node *k*.

In the beginning, these weights need to be initialized. This initialization process is influenced by two hyperparameters: average rule length ( $\bar{l}$ ) and initialization probability (*p*), where $\bar{l}$ only affects the number of weights that are set to one in the first layer. Here we use the additional information which literals belong to the same attribute to avoid immediate contradictions within the first conjunction. Let |A| be the number of attributes, then each attribute is selected with the probability $\bar{l}/|A|$ so that on average for $\bar{l}$ literals of different attributes the corresponding weight will be set to true. In the remaining layers, the weights are set to true with the probability *p*. Additionally, at least one outgoing weight from each node will be set to true to ensure connectivity. This implies that, regardless of the choice of *p*, all the weights in the last layer will always be initialized with true because there is only one output node. Note that, as a consequence, shallow DNF-structured networks will not be influenced by the choice of *p*, since they only consist of the first layer influenced by $\bar{l}$ and the last layer initialized with true.

### 3.3 Prediction

The prediction of the network can be efficiently computed using binary matrix multiplications (⊚). In each layer *i*, the input features *A* ^(*i*)^ are multiplied with the corresponding weights *W* ^(*i*)^ and aggregated at the receiving node in layer *i* + 1. If the aggregation is disjunctive, this directly corresponds to a binary matrix multiplication. Each product of an input feature *a* ^(*i*)^ and its corresponding weight *w* ^(*i*)^ is either true or false, and the summation of these products is set to true for any sum larger than zero, i.e., if any rule fires. Furthermore, according to De Morgan’s law, *a* ∧ *b* = *¬*(*¬a* ∨*¬b*) holds. This means that binary matrix multiplication can be used also in the conjunctive case, provided that the inputs and outputs are negated before and after the multiplication. Because of the alternating sequence of conjunctive and disjunctive layers, binary matrix multiplications and negations are also always alternated when passing data through the network, so that a binary matrix multiplication followed by a negation can be considered as a NOR-node. Thus, the activations *A* ^(*i*+1)^ can be computed from the activations in the previous layers as

$$A^{\left(i+1\right)}\leftarrow {\tilde{A}}^{\left(i\right)}⊚W^{\left(i\right)}$$ (1)

where $\tilde{X}=J-X$ denotes the element-wise negation of a matrix *X* (*J* denotes a matrix of all ones). Hence, internally, we do not distinguish between conjunctive and disjunctive layers within the network, but have a uniform network structure consisting only of NOR-nodes. However, for the sake of the ease of interpretation, we chose to represent the networks as alternating AND and OR layers.

In the first layer, we have the choice whether to start with a disjunctive layer or a conjunctive one, which can be controlled by simply using the original input vector (*A* ^(0)^ = x) or its negation ( $A^{\left(0\right)}=\tilde{x}$ ) as the first layer. Also, if the last layer is conjunctive, an additional negation must be performed at the end of the network so that the output has the same “polarity” as the target values. In our experiments, we always start with a conjunctive and end with a disjunctive layer. In this way, the rule networks can be directly converted into conjunctive rule sets.

**FIGURE 3:**
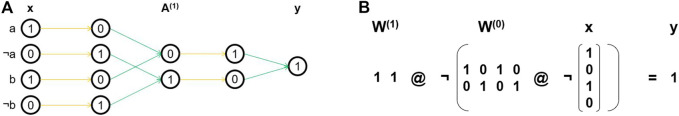
Example network and binary matrix multiplications for the parity concept with two variables, *y* = (*a* ∧ *b*) ∨ (*¬a* ∧*¬b*). Orange connections are logical negations (*¬*), green edges correspond to binary matrix multiplications (@). **(A)** Activations processed through the network. **(B)** matrix multiplication.

Figure 3 illustrates an example prediction for the parity concept with two variables, *a* and *b*, and two rules *y* = *a* ∧ *b* and *y* = *¬a* ∧*¬b*. Negations are marked by *¬* and orange arrows, Boolean matrix multiplications by @ and green arrows. In Figure 3A, the computed negations and activations for an input *x* = {*a*, *b*} are stated within the nodes. The corresponding weight matrices and the complete prediction formula are shown in Figure 3B.

### 3.4 Training

Following Beck and Fürnkranz (2020), we implement a straight-forward mini-batch based greedy optimization scheme. While the number, the arrangement and the aggregation types of the nodes remain unchanged, the training process will flip the weights of the network to optimize its outcome. Flipping a weight from 0 to 1 (or vice versa) can be understood to be a single addition (or removal) of a literal to the conjunction or disjunction encoded by the following node.

Algorithm 1 Deep Rule Network Training, fit()-method

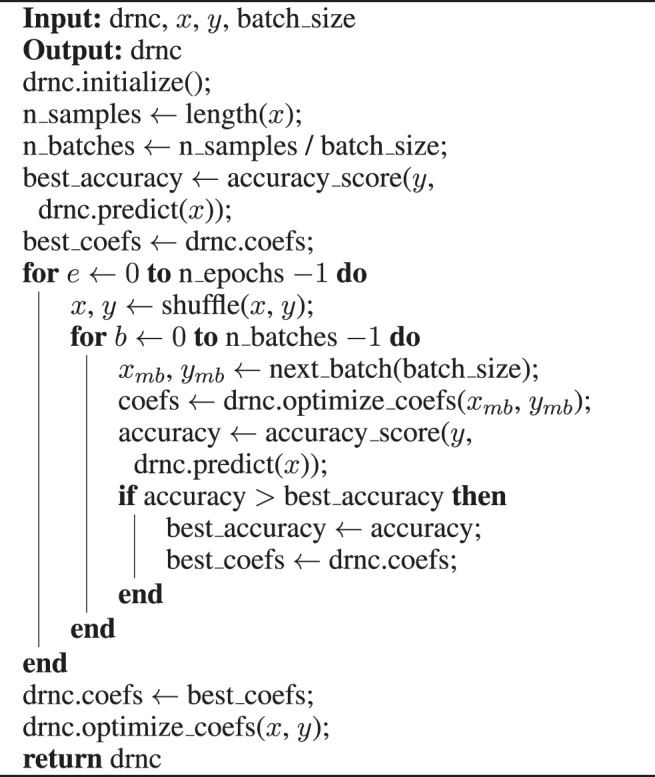

Algorithm 2 Deep Rule Network Training, optimize_coefs()-method

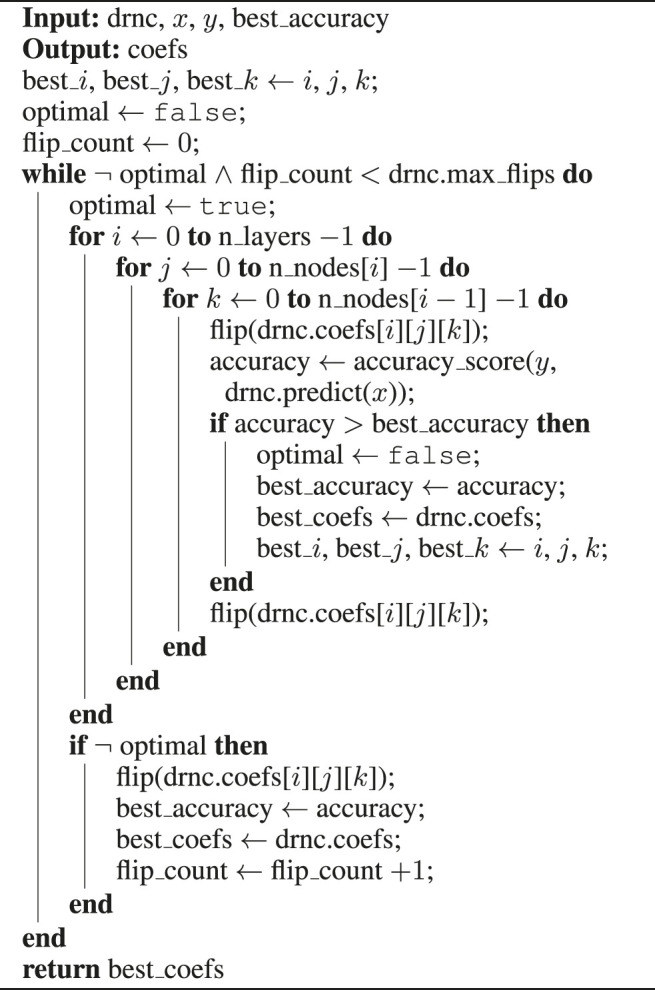

A detailed pseudo-code of the training process is shown in Algorithms 1 and 2. Given a deep rule network classifier *drnc*, training samples *x*, their correct targets *y* and an appropriate batch size for the training set, Algorithm 1 shows a naïve greedy approach to fit the network to the training data. After the initialization, the base accuracy on the complete training set and the initial weights are stored and subsequently updated every time when the predicted accuracy on the training set exceeds the previous maximum after processing a mini-batch of training examples. However, the predictive performance does not necessarily increase monotonically, since the accuracy is optimized not on the whole training set, but on a mini-batch. For all layers and nodes, possible flips are tried and evaluated, and the flip with the biggest improvement of the accuracy on the current mini-batch is selected. These greedy adjustments are repeated until either no flip improves the accuracy on the mini-batch or a maximum number of flips is reached, which ensures that the network does not overfit the mini-batch data.

When all mini-batches are processed, the procedure is repeated for a fixed number of epochs. Only the composition of the mini-batches is changed in each epoch by shuffling the training data before proceeding. After all epochs, the weights of the networks are reset to the optimum found so far, and a final optimization on the complete training set is conducted to eliminate any overfitting on mini-batches. The returned network can then be used to predict outcomes of any further test instances.

## 4 Experiments

In this section we present the results of differently structured rule networks on both artificial and real-world UCI datasets, with the goal of investigating the effect of differences in the depth of the networks. We first describe the artificial datasets (*Artificial Datasets*), then some preliminary experiments that helped us to focus on suitable network structures and hyperparameters (*Hyperparameter Tuning*), and finally discuss the main results on the artificial and real datasets. The code and the datasets are available in a public repository^2^.

### 4.1 Artificial Datasets

As many standard UCI databases can be solved with very simple rules (Holte, 1993), we generated artificial datasets with a deep structure that we know can be represented by our network. An artificial dataset suitable for our greedy optimization algorithm should not only include intermediate concepts which are meaningful but also a strictly monotonically decreasing entropy between these concepts, so that they can be learned in a stepwise fashion in successive layers. One way to generate artificial datasets that satisfy these requirements is to take the output of a randomly generated deep rule network. Subsequently, this training information can be used to see whether the function encoded in the original network can be recovered. Note that such a recovery is also possible for networks with different layer structures. In particular, each of the logical functions encoded in such a deep network can, of course, also be encoded as a DNF expression, so that shallow networks are not in an a priori disadvantage (provided that their hidden layer is large enough, which we ensure in preliminary experiments reported in *Hyperparameter Tuning*).

We use a dataset of ten Boolean inputs named *a* to *j* and generate all possible 2^10^ combinations as training or test samples. These samples are extended by the ten negations *¬a* to *¬j* via one-hot-encoding and finally passed to a funnel-shaped deep rule network with *n* = 5 and s = [32, 16, 8, 4, 2]. The weights of the network are set by randomly initializing the network and then training it on two randomly selected examples, one assigned to the positive and one to the negative class, to ensure both a positive and negative output is possible. If the resulting ratio of positively predicted samples is still less than 20*%* or more than 80*%*, the network is reinitialized with a new random seed to avoid extremely imbalanced datasets.

**FIGURE 4:**
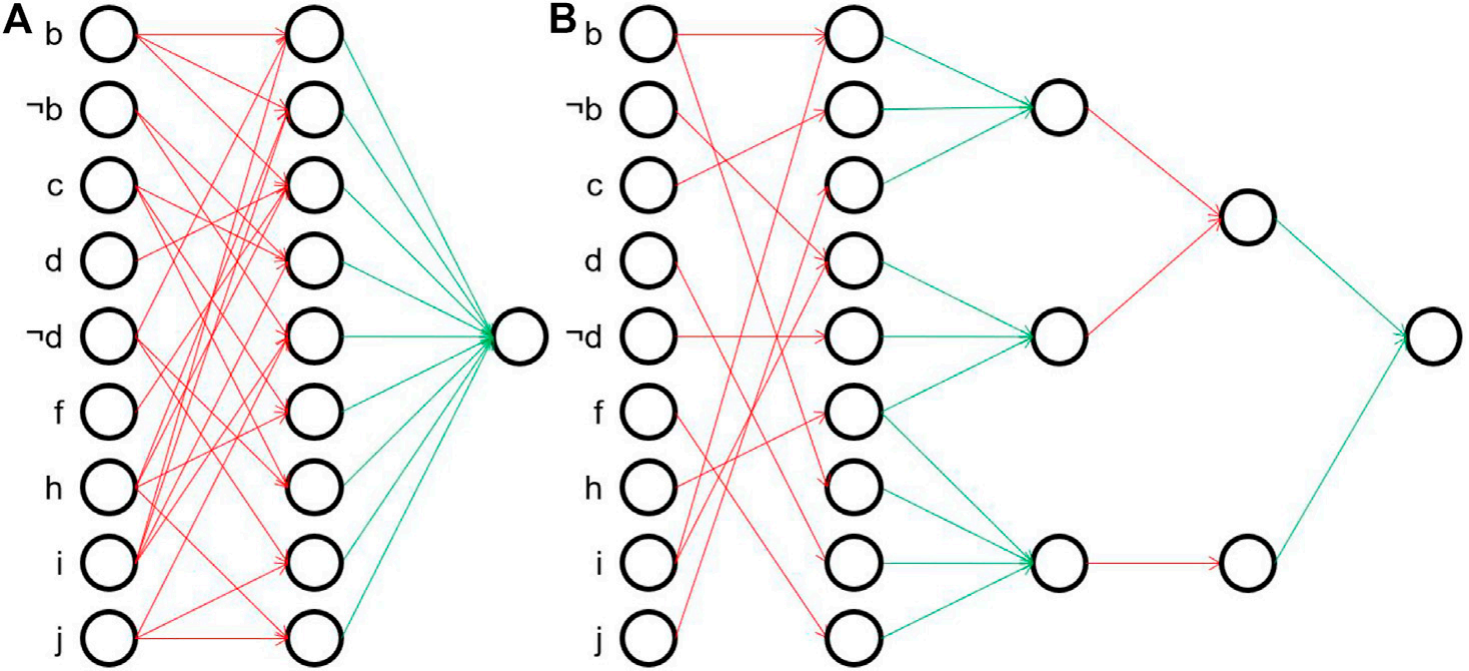
Example networks of Eq. 2, 3. For a better overview, the input nodes not influencing the final concept are removed. Red connections are logical ANDs, green edges correspond to logical ORs. **(A)** Shallow example. **(B)** Deep example.

An example concept for a generated dataset is shown in Figure 4. Thinking of a rule network, circles represent nodes and are connected by an arrow if and only if the corresponding weight is true. Note that many nodes and weights are irrelevant, and are not shown, e.g., neither *a* nor *¬a* have an influence on the generated output. The flat representation shown in Figure 4A corresponds to the following DNF expression^3^:

(b∧¬d∧i)∨(b∧h∧i)∨(b∧d∧f∧h)∨(¬b∧c∧i)∨(¬b∧i∧j)∨(c∧h)∨(c∧¬d)∨(¬d∧j)∨(h∧j) (2)

It can be clearly seen that the rules in this simplified formula share some common features, indicating intermediate concepts in subsequent layers which are combined in the end. A more compact representation of the same concept using a hierarchical structure is:

$$\left(\left(\left(b\wedge i\right)\vee c\vee j\right)\wedge \left(\left(\neg b\wedge i\right)\vee \neg d\vee h\right)\right)\vee \left(b\wedge d\wedge f\wedge h\right)$$ (3)

The second representation only needs 11 aggregations (6 AND, 5 OR) in comparison to 23 aggregations (15 AND, 8 OR) in the first one. This is also reflected in the number of weights set to true, i.e. the number of arrows in Figure 4 (33 in Figure 4A vs. 26 in Figure 4B). In contrast, when training the deep rule network, we must learn at least 180 + 27 + 6 + 2 = 215 binary weights correctly, while for the shallow one already 180 + 9 = 189 would be sufficient. Note that we included here the eleven nodes in the input layer which are absent in the formulas, but whose weights nevertheless have to be learned by both networks. Looking at the figures, it is also noticeable that even though both networks have sparse weight matrices, the ones of the hierarchical network are even more sparse which makes it almost shaped like a tree. In the following experiments, we evaluate which of these two representations is easier to learn approximately.

### 4.2 Hyperparameter Tuning

Before the main experiments, we conducted a few preliminary experiments on three of the artificial datasets to set suitable default values for the hyperparameters of the networks. One of these hyperparameters also known from neural networks is the number of epochs (*n*_*epochs*). By definition (Algorithm 1), the accuracy monotonically increases with a higher number of epochs while at the same time the training time rises as well. After five epochs, the performance no longer rises remarkably, so this value seems as a good trade-off between performance and training time.

The second hyperparameter *batch*_*size* affects these two measures as well. After tests using the number of instances as *batch*_*size* and others skipping the final optimization in Algorithm 1 on the full batch, we notice that using a combination of mini-batches and full batches performs better than either of the two individual batch variants. For the artificial datasets, a *batch*_*size* of 50 is suitable. Finally, the limitation of iterations per mini-batch by *max*_*flips* also influences both the accuracy and training time. However, in case of noise-free artificial data, we can leave *max*_*flips* unbounded to achieve the optimal performance.

The experiments also showed no clear advantages or disadvantages between a conjunctive or disjunctive first layer, so in the following experiments we focus on networks starting with a conjunctive layer which offer the biggest similarity and best comparability to models learned from classic rule learners. Furthermore, we dispense with a separate optimization of the last layer like in (Beck and Fürnkranz, 2020), as this did not result in any improvement in performance.

For the remaining hyperparameters, we tried to find appropriate settings by performing a grid search on 20 artificial datasets. The hyperparameters to be optimized are the average rule length $\bar{l}$ , the initialization probability *p*, the number *n* and the sizes *s*_*i*_ of hidden layers. The other hyperparameters are set to the default values stated above, except that only a single epoch is used in order to speed up the grid search. This will have a negative effect on the performance in general, but should not significantly change the ranking of the different networks.

Table 1 shows the hyperparameters that we compared in a grid search. For the deep networks, we set *n* to values from 3 to 5. On the one hand, this guarantees that they contain at least two conjunctive and two disjunctive layers to map a wide variety of hierarchical concepts effectively. On the other hand, it still permits that the values of *s*_*i*_ can be set to values bigger than 10 while maintaining a reasonable training time with the naïve greedy algorithm. We create two different networks for each of the three values of *n* and set the values *s*_*i*_ so that *s*_*i*_ ≥ 2*s*_*i*+1_, resulting in a smaller network and a bigger one containing 1.5 to 3.5 times as many weights that can be adapted. For shallow networks, *n* is by definition set to 1. To ensure that these networks have approximately the same expressive power as the corresponding deep networks, we set *s*_1_ so that the total number of weights in both network types is roughly the same. Additionally, we try a very high number of *s*_1_ = 500 rules to estimate if a very big single layer can improve the accuracy remarkably. For the average initialized rule length $\bar{l}$ we will use the integer values from one to three for deep networks and from one to seven for shallow ones. We assume that in shallow networks a higher value of $\bar{l}$ is required, while in deep networks the intermediate concepts can be combined in successive layers. The numerical deficit of deep network test cases caused by $\bar{l}$ is compensated by the additional hyperparameter *p*, where we use three values between O.O25 and O.125. Therefore, in total, the accuracy of the deep network will be mapped to three dimensions *n*|*s*_*i*_, $\bar{l}$ and *p* and the accuracy of the shallow network to only two dimensions *s*_1_ and $\bar{l}$ .

**TABLE 1:** Hyperparameters for deep and shallow networks.

	Deep	shallow
	5: (72, 36, 12, 6, 2), (32, 16, 8, 4, 2)	
*n*, *s*_*i*_	4: (36, 12, 6, 2), (16, 8, 4, 2)	1: 10, 20, 50, 100, 200, 500
	3: (12, 6, 2), (8, 4, 2)
l	1, 2, 3	1, 2, 3, 4, 5, 6, 7
*P*	0.025, 0.075, 0.125	‐

**FIGURE 5:**
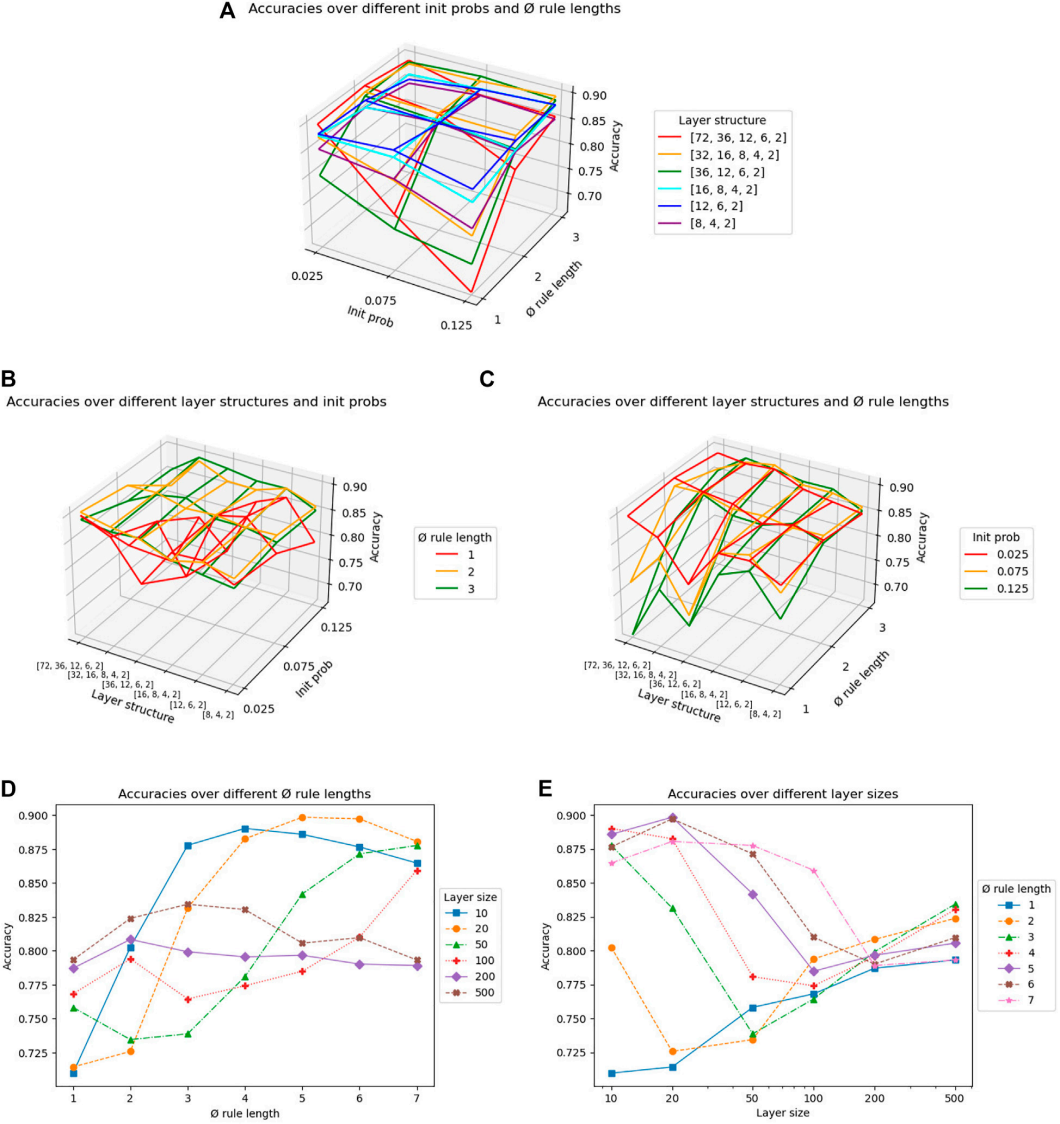
Grid search on the hyperparameters average rule length $\bar{l}$ , initialization probability *p*, number *n* and sizes *s*_*i*_ of hidden layers. The first three plots show accuracies for deep rule networks with different combinations of **(A)** *p* and $\bar{l}$ , **(B)** *n*|*s*_*i*_ and $\bar{l}$ and **(C)** *n*|*s*_*i*_ and *p*. The last two show accuracies for shallow rule networks with different **(D)** $\bar{l}$ and **(E)** *s*_1_.

The results of the grid search are shown in Figure 5. The optimal hyperparameter setting for deep networks with an accuracy of 0.9073 is s = (72, 36, 12, 6, 2), $\bar{l}=2$ and *p* = 0.025. However, we notice in Figure 5A that the red curve of the largest structure s = (72, 36, 12, 6, 2) not only contains the maximum, but also the minimum accuracy with $\bar{l}=1$ and *p* = 0.125. While in general the combination of a lower $\bar{l}$ and a higher *p* decreases the accuracy, this effect seems to be stronger the bigger the network structure is. Despite the higher sensitivity, the larger layer structures provide better maximum accuracies than the smaller ones, as can be seen in the upper left corner of the graph. When comparing the graphs for different values of $\bar{l}$ in Figure 5B, it is noticeable that, with only few exceptions, the red curve for $\bar{l}=1$ lies below the other two curves. The same can be observed in Figure 5C, here for the green curve for *p* = 0.125. Combinations of other values of $\bar{l}$ and *p* provide good accuracies regardless of the layer structure.

For shallow networks, we can see a high correlation between *s*_1_ and $\bar{l}$ in Figure 5D. The graphs (except of the red one) resemble a downward-opening parabola, so that the accuracy becomes lower and lower the greater the deviation from this optimal value. Thereby applies that the smaller the layer size, the larger is the optimal value of $\bar{l}$ , e.g., 5 for 20 and 2 for 200. Finally, Figure 5E shows that, contrary to the deep networks, the sensitivity to $\bar{l}$ decreases with the size of the shallow network. The optimal accuracy of 0.8984 is reached when the shallow network hyperparameters are set to *s*_1_ = 20 and $\bar{l}=5$ .

Based on the above results, we will only use three network versions for the main experiments reported in the following sections. As a candidate for shallow networks, we take the best combination of *s*_1_ = 20 and $\bar{l}=5$ . For the deep networks, however, we will choose the second-best network s = (32, 16, 8, 4, 2) combined with $\bar{l}=2$ and an averaged *p* = 0.05, since it is almost ten times faster than the best deep network while still reaching an accuracy over 0.895. The third network is chosen as an intermediate stage between the first two: s = (32, 8, 2) combined with $\bar{l}=3$ and *p* = 0.05. While still being a deep network, the learned rules can be passed to the output layer a little faster. In the following, we will refer to these (deep) rule network classifiers based on their number of layers, i.e. DRNC(5) for s = (32, 16, 8, 4, 2), DRNC(3) for s = (32, 8, 2) and RNC for *s*_1_ = 20. For computational reasons, all of the reported results were estimated with a 2-fold cross validation. While this may not yield the most reliable estimate on each individual dataset, we nevertheless get a coherent picture over all 20 datasets, as we will see in the following.

### 4.3 Results on Artificial Datasets

In the main experiments, we use a combination of 15 artificial datasets with seeds we already used in a prior hyperparameter grid search and five artificial datasets with new seeds to detect potential overfitting on the first datasets. All datasets are tested using five epochs, a batch size of 50 and an unlimited number of flips per batch. We also ensured for all of the generated datasets that the DNF concept does not contain more than 20 rules, so that it can theoretically also be learned by the tested shallow network with *s*_1_ = 20 (and therefore also for the two deep networks, since their first layer is already bigger).

**FIGURE 6:**
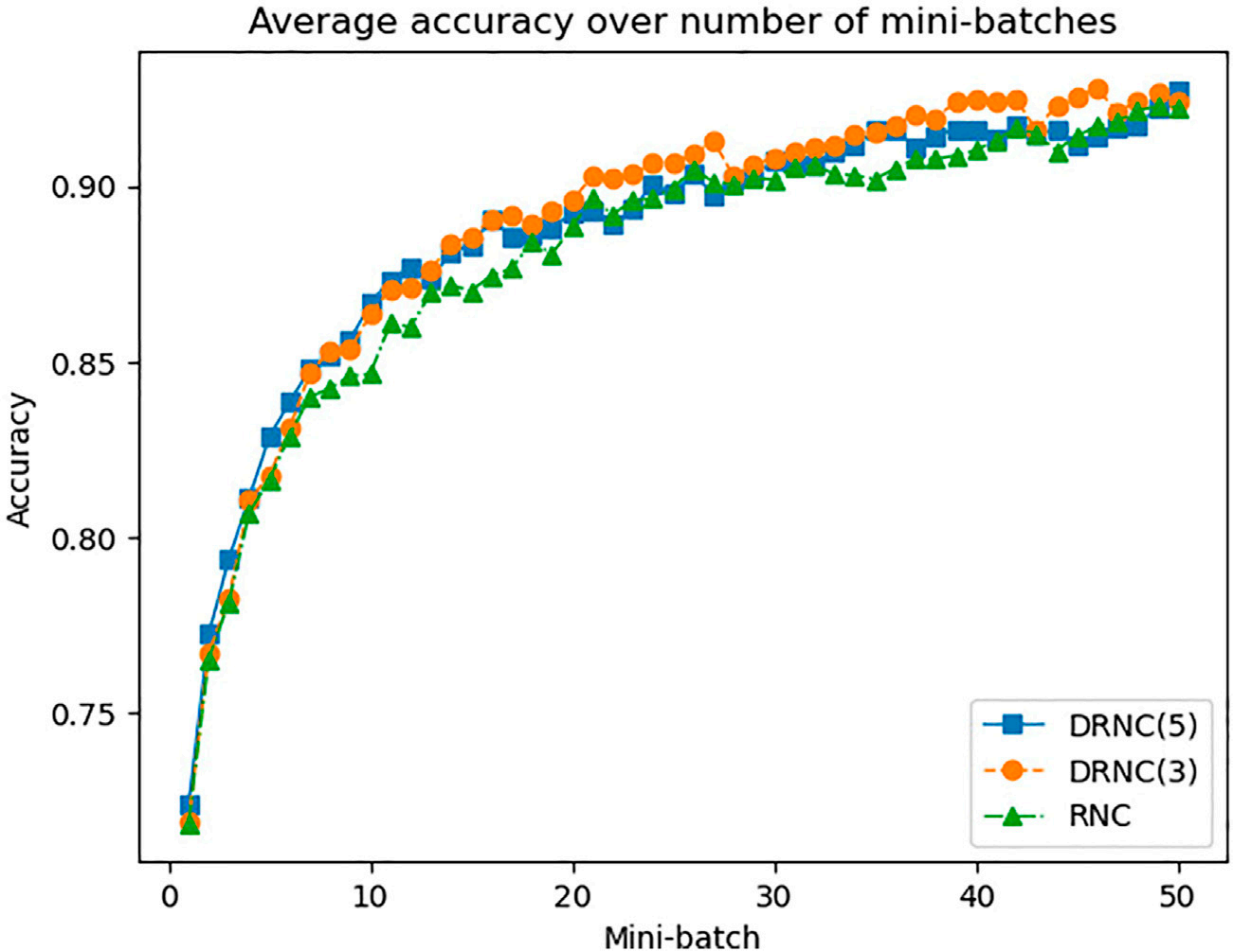
Average accuracy of rule network with 1/3/5 layers on training dataset.

Figure 6 shows the development of the accuracies on the training set averaged on all 20 datasets over the number of processed mini-batches, whereby after every ten mini-batches a new epoch starts. The base accuracy before processing the first mini-batch and after the full batch optimization are omitted. We can see that the deep networks not only deliver higher accuracies but they also converge slightly faster than the shallow one. The orange curve of DRNC(3) runs a little higher than the blue one of DRNC(5), whereas the green curve of RNC has some distance to them, especially during the first two epochs.

Table 2 shows the accuracies of the three networks. For each dataset, the best accuracy of the three network classifiers is highlighted in bold, the best accuracy including RIPPER and CART in italic. When comparing the rule network approaches, we can see a clear advantage for the two deep networks both when considering the average accuracy and the amount of highest accuracies. The results clearly show that the best performing deep networks outperform the best performing shallow network in all but four of the 20 generated datasets. Both the average rank and the average accuracy of the deep networks is considerably better than the corresponding values for RNC. This also holds for pairwise comparisons of the columns (DRNC(5) vs RNC 15:5, DRNC(3) vs RNC 15:5).

**TABLE 2:** Accuracies on artificial datasets. Rule network with 1/3/5 layers vs RIPPER vs CART. The best accuracy of the rule networks is marked in bold, the overall best accuracy per dataset is marked in italic.

seed	% (+)	DRNC(5)	DRNC(3)	RNC	RIPPER	CART
5	0.4453	0.958	**0.9863**	0.9531	0.9805	0.9844
16	0.7959	0.9639	**0.9707**	0.9629	0.9766	0.9551
19	0.6562	***1***	0.9902	0.9746	1	1
24	0.584	0.9053	0.9043	**0.916**	0.9463	0.9404
36	0.6943	0.8828	***0.9209***	0.9043	0.8867	0.9111
44	0.7939	**0.9629**	0.9551	0.9326	0.9482	0.9697
53	0.6055	**0.9805**	**0.9805**	0.9775	0.9746	0.9824
57	0.7705	**0.9824**	0.9736	0.9639	0.9951	0.9902
60	0.7715	0.9443	**0.9453**	0.9209	0.958	0.9883
65	0.5312	**0.9854**	0.9688	0.9414	0.9961	0.9922
68	0.5654	0.9248	0.9443	**0.9619**	0.9688	0.9355
69	0.6924	0.9551	**0.9658**	0.9199	0.9795	0.9717
70	0.6338	0.9014	0.9062	**0.9229**	0.9111	0.8984
81	0.5684	0.9004	**0.9131**	0.8857	0.9248	0.9756
82	0.7188	0.9941	**0.998**	0.9717	1	1
85	0.5312	***1***	0.998	0.9736	1	1
89	0.6084	0.8926	0.9434	**0.9629**	0.9502	0.9395
107	0.6172	**0.8965**	0.873	0.8643	0.9043	0.9277
112	0.7549	**0.9346**	0.9248	0.9189	0.9082	0.9561
118	0.5957	**0.9688**	0.9414	0.9434	0.9736	0.9688
Ø Accuracy		0.9467	0.9502	0.9386	0.9591	0.9644
Ø Rank		1.775	1.725	2.5	–	–

**FIGURE 7:**
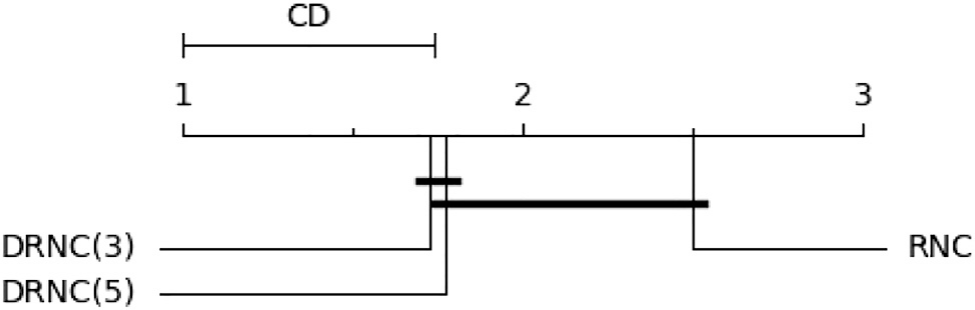
Critical distance diagram for the rule networks on the artificial datasets with a significance level of 95*%*. Learners that are not statistically different from one another are connected.

The Friedman test for the ranks yields a significance of more than 95%. A subsequent Nemenyi Test delivers a critical distance of 0.741 (95%) or 0.649 (90%), which shows that DRNC(3) and RNC are significantly different on a level of more than and DRNC(5) and RNC on a level of more than 90*%*. The corresponding critical distance diagram (CD = 0.741) is shown in Figure 7. We thus find it safe to conclude that deep networks outperform shallow networks on these datasets.

In the two right-most columns of Table 2 we also show a comparison to the state-of-the-art rule learner RIPPER (Cohen, 1995) and the decision tree learner CART (Breiman et al., 1984) in Python implementations using default parameters.^4^ We see that all network approaches are outperformed by the RIPPER and CART classifiers with default setting. The difference between RIPPER and DRNC(3) is approximately the same as the difference between DRNC(3) and RNC. However, considering that we only use a naïve greedy algorithm, it could not be expected (and was also not our objective) to be able to beat state-of-the-art rule learner. In particular, the runtime is far from state-of-the-art, since already for the shallow network 30 seconds are needed per dataset and up to 3 minutes for the deep networks (in comparison to less than a second for RIPPER and CART). Furthermore, the results also confirm that shallow rule learners (of which both RIPPER and CART are representatives) had no disadvantage by the way we generated the datasets.

### 4.4 Results on UCI Datasets

For an estimation how the rule networks perform on real-world datasets, we select nine classification datasets (*car-evaluation*, *connect-4*, *kr-vs-kp*, *monk* one to three, *mushroom*, *tic-tac-toe* and *vote*) from the UCI Repository (Dua and Graff, 2017). They differ in the number of attributes and instances, but have in common that they consist only of nominal attributes. *Car-evaluation* and *connect-4* are actually multi-class datasets and are therefore converted into the binary classification problem whether a sample belongs to the most frequent class or not. Of all binary classification problems, the networks to be tested treat again the more common class as the positive class and the less common as the negative class, except for the *monk* datasets whereby the positive class is set to 1. As with the artificial datasets, we additionally compare the performance of the networks to RIPPER and CART, and again all accuracies are obtained via 2-fold cross validation. In case a random initialization did not yield any result (i.e., the resulting network classified all examples into a single class), we re-initialized with a different seed (this happened once for both deep network versions).

The results are shown in Table 3. We can again observe that both deep networks outperform the shallow network RNC. Of all rule networks, DRNC(5) provides the highest accuracy on the *connect-4*, *monk-1*, *monk-3*, *mushroom* and *vote* datasets, whereas DRNC(3) performs best on *car-evaluation* and *monk-2* and RNC on *kr-vs-kp* and *tic-tac-toe*. The comparison to RIPPER and CART is again clearly in favor of these state-of-the-art algorithms. We will analyze some of the rule networks in the following subsection.

**TABLE 3:** Accuracies on real-world datasets. Rule network with 1/3/5 layers vs RIPPER vs CART. The best accuracy of the rule networks is marked in bold, the overall best accuracy per dataset is marked in italic.

Dataset	% (+)	DRNC(5)	DRNC(3)	RNC	RIPPER	CART
car-evaluation	0.7002	0.8999	**0.9022**	0.8565	0.9838	0.9821
connect-4	0.6565	**0.7728**	0.7712	0.7597	0.7475	0.8195
kr-vs-kp	0.5222	0.9671	0.9643	**0.9725**	0.9837	0.989
monk-1	0.5000	***1***	0.9982	0.9910	0.9478	0.8939
monk-2	0.3428	0.7321	**0.7421**	0.7139	0.6872	0.7869
monk-3	0.5199	0.9693	0.9603	0.9567	0.9386	0.9729
mushroom	0.784	***1***	0.978	0.993	0.9992	1
tic-tac-toe	0.6534	0.8956	0.9196	**0.9541**	1	0.9217
Vote	0.6138	**0.9655**	0.9288	0.9264	0.9011	0.9287
Ø Rank		1.556	2	2.444	–	–

### 4.5 Interpretation of Learned Models

While the results in the previous two subsections indicate that deep rule networks perform better than shallow ones on both artificial and UCI datasets, we now try to find the reason for this by analyzing the learned models of all three rule network candidates. We investigate into the models for the artificial dataset generated by seed 44 and the first and third monk dataset since for those datasets the deep rule networks did not only outperform their shallow counterpart but also RIPPER.

For the artificial dataset, we convert the ground truth and the learned networks into equivalent DNF formulas, i.e., flat rules, which allows us to more easily compared the learned concepts with the ground truth. Table 4 shows the results, where each line corresponds to one rule, with similar rules being grouped together. We notice that all three learned models are smaller than the original one (8–10 rules instead of 16), which is mainly due to the rules in the last five rows, that are combined to a simpler and only marginally worse rule *f* ∧*¬i* → true by all rule networks. For DRNC(3) and RNC, this is also the case for some other rules, with RNC having the biggest generalization with the simple rule *¬g* → true. In contrast, DRNC(5) learns more specific rules, and five out of ten are also part of the DNF of the ground truth (highlighted in bold in Table 4).

**TABLE 4:** DNF models of deep and shallow rule networks for artificial dataset with seed 44. Each row shows a rule (body), which are disjuncted for the final model. Rules that are also contained in the ground truth are marked in bold.

Ground Truth	DRNC(5)	DRNC(3)	RNC
–	–	–	*¬a* ∧*¬b* ∧*¬e* ∧*¬i* ∧*¬j*
*¬b* ∧ *c* ∧*¬i*	–	–	–
*a* ∧*¬e* ∧*¬g*	***a ∧¬e ∧¬g***	–	*¬g*
*¬b* ∧*¬g* ∧ *i*	*¬b* ∧*¬g*	*¬b* ∧*¬g*	–
*¬b* ∧*¬d* ∧*¬g*	–	–	–
*f* ∧*¬g*	***f ∧¬g***	***f ∧¬g***	–
	*¬g* ∧*¬h* ∧*¬j*	–	–
*c* ∧*¬f* ∧ *g*	***c ∧¬f ∧ g***	*c* ∧*¬f*	*c* ∧*¬f*
*c* ∧*¬f* ∧*¬j*	–	–	–
	*¬a* ∧ *c* ∧ *h*	*c* ∧ *h*	*¬a* ∧ *c*, *c* ∧ *h*
*c* ∧ *h* ∧ *i*	***c ∧ h ∧ i***	–	–
*¬c* ∧*¬e* ∧ *h*	*¬e* ∧ *h*	*¬e* ∧ *h*	*¬e* ∧ *h*
*¬c* ∧*¬g* ∧*¬h*	***¬c ∧¬g ∧¬h***	*¬g* ∧*¬h*	–
*d* ∧*¬e* ∧*¬i* ∧*¬j*	–	***d ∧¬e ∧¬i ∧¬j***	***d ∧¬e ∧¬i ∧¬j***
*¬a* ∧ *f* ∧*¬i*	*f* ∧*¬i*	*f* ∧*¬i*	*f* ∧*¬i*
*b* ∧ *f* ∧*¬i*	–	–	–
*¬d* ∧ *f* ∧*¬i*	–	–	–
*f* ∧*¬h* ∧*¬i*	–	–	–
*f* ∧*¬i* ∧*¬j*	–	–	–

We further investigate into the models learned by the rule networks in terms of interpretability. The shallow network consists of some redundant rules that, however, can easily be converted to the DNF presented in Table 4. When looking at the full model of the two deep networks, it is hard to see which concepts of the first layer contribute to the final prediction because of hidden branches and redundancies. After manually simplifying the model of DRNC(3), we notice that the two last layers are not used anymore and we therefore obtain a model similar to the one of the shallow network. While the deep structure seems to promote the finding of a suitable model by offering multiple paths to generate a correct prediction, the resulting model is nevertheless limited to one of these paths and effectively delivers a shallow model that is, however, hard to detect and less interpretable.

**FIGURE 8:**
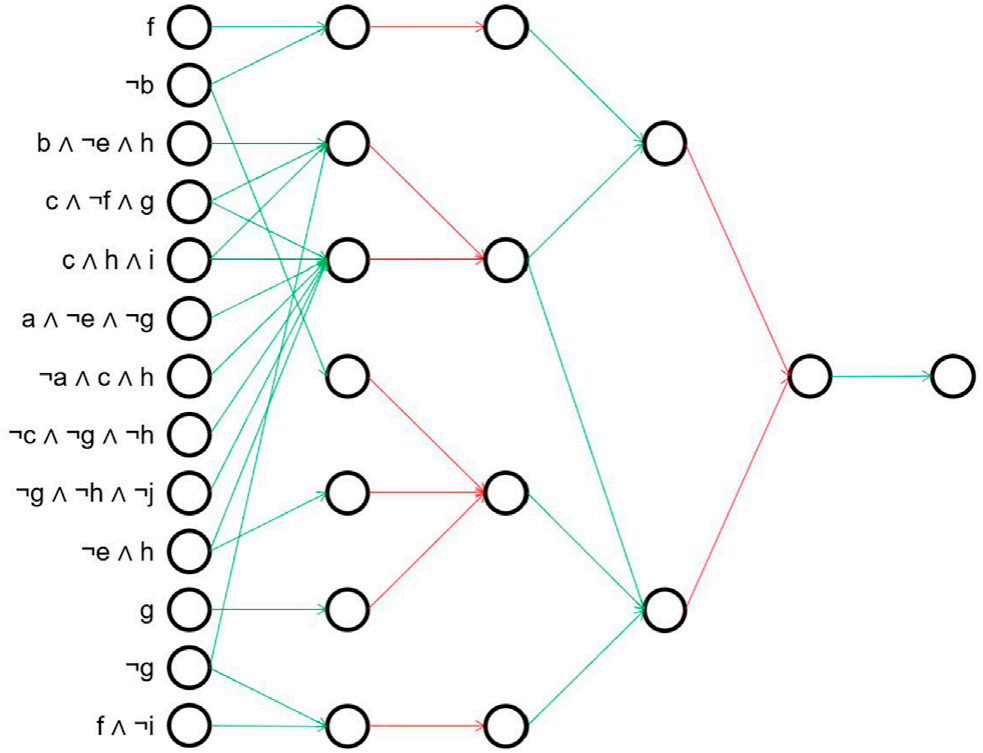
Simplified model of DRNC(5) for artificial dataset with seed 44. For a better overview, the first layer is hidden and instead the resulting rules are listed directly. Red connections are logical ANDs, green edges correspond to logical ORs.

For the model learned by DRNC(5), the simplified structure is still deep. Figure 8 illustrates this by replacing the first AND-layer with the resulting logical terms, and showing how they are further combined in subsequent layers. In contrast to the model of DRNC(3), even after the removal of redundant concepts and subtrees the structure remains hard to interpret. While some of the underlying concepts in the left-most layer are passed unchanged through the network (e.g. node 4: *c* ∧*¬f* ∧ *g*), others make actually use of the deep structure and are further combined in subsequent layers (e.g. node 1 and 12 are merged to *f* ∧*¬g*). Some of the aggregations are unnecessarily cumbersome: The concept *¬e* ∧ *h* in node 10 is first conjuncted with nodes 2 and 11, but subsequently disjuncting with nodes 2, 3 and 12 results again in the original concept *¬e* ∧ *h*. To conclude, the learned model is reasonable, but not nearly as compact as it could be which requires a deeper analysis to understand the network.

A similar behavior can be recognized for the first monk dataset. Its target concept is *a*1 = *a*2 ∨ *a*5 = 1, which requires four concepts *a*1 = 1 ∧ *a*2 = 1, *a*1 = 2 ∧ *a*2 = 2, *a*1 = 3 ∧ *a*2 = 3 and *a*5 = 1 to be identified by the tested rule learners. All three rule networks are able to detect these concepts but with minor differences. The shallow rule network RNC learns three of the four concepts directly and uses a combination of two rules for the remaining one. Moreover, the learned model consists of three more rules that are not redundant, but cover additional examples in the test set (but not in the training set). DRNC(3) learns three of the four concepts directly as well. The remaining concept is split into two subconcepts *a*1 = 1 and *a*2 = 1, that are combined in the additional conjunctive layer in the deep structure. As for the RNC model, some additional rules are left that cover a few spurious cases and cause the small error rate during testing. Last, the DRNC(5) model achieves a perfect accuracy by detecting three of the four concepts directly and constructing the remaining one like the DRNC(3) network in the second conjunctive layer.

The results are similar for the third monk dataset with the target concept *a*5 = 3 ∧ *a*4 = 1 ∨ *a*5 ≠ 4 ∧ *a*2 ≠ 3. The inequations have to be learned again by multiple rules, but this time the accuracies are worse for all networks because of the noise that had been added to the data. Both the model of RNC and the simplified model of DRNC(3) are in DNF form, whereas the simplified model of DRNC(5) still makes use of the deep structure. We assume that resulting from this, the DRNC(5) model generalizes better, since in the DRNC(3) and in particular in the RNC model there are additional rules left which overfit the training data. A deep structure seems to provide more and better options to prune the model, since flips in layers close to the output have effects on possibly multiple inputs (this would of course have to be checked in more detail in separate experiments). However, we also note that this accuracy gain comes at the cost of interpretability.

So, in summary, we can observe that useful structures emerge in the deep networks, which are, however, not easily interpretable. We are currently working on employing methods for logic minimization to reduce the size of a learned deep network in order to increase their interpretability.

## 5 Conclusion

The main objective of this work was to study the question whether deep rule networks have the potential of outperforming shallow DNF rule sets, even though, in principle, every concept can be represented as DNF formula. As there is no sufficiently competitive deep rule learning algorithm, we proposed a technique how deep and shallow rule networks can be learned and thus effectively compared in a uniform framework, using a network approach with a greedy, mini-batch based optimization algorithm. For both types of networks, we find good hyperparameter settings that allow the networks to reach reasonable accuracies on both artificial and real-world datasets, even though the approach is still outperformed by state-of-the-art learning algorithms such as RIPPER and CART.

Our experiments on both artificial and real-world benchmark data indicate that deep rule networks outperform shallow networks. The deep networks obtain not only a higher accuracy, but also need less mini-batch iterations to achieve it. Moreover, in preliminary experiments in the hyperparameter grid search, we have seen indications that the deep networks are generally more robust to the choice of the hyperparameters than shallow networks. On the other hand, we also had some cases on real-world data sets where deep networks failed because a poor initialization resulted in indiscriminate predictions. The current approach is also limited to binary classification problems with nominal attributes.

Overall, we interpret these results as evidence that an investigation of deep rule structures is a promising research goal, which we hope could yield a similar boost in performance in inductive rule learning as could be observed by moving from shallow to deep neural networks. However, this goal is still far ahead.

## 6 Future Work

In this work, it was not our goal to reach a state-of-the-art predictive performance, but instead we wanted to evaluate a very simple greedy optimization algorithm on both shallow and deep networks, in order to get an indication on the potential of deep rule networks. Nevertheless, several avenues for improving our networks have surfaced, which we intend to explore in the near future.

One of the main drawbacks of the presented deep rule networks is the extremely high runtime due to the primitive flipping algorithm. A single flip needs a recalculation of all activations in the network, even if only a few them will be affected by this flip whereby the matrix multiplication could be minimized considerably. Conversely, this knowledge can be used to find a small subset of flips that affects a certain activation. On the other hand, the majority of possible flips does not have any effect on this activation or the accuracy at all. This effect will typically remain unchanged after a few more flips are done. Therefore, an exhaustive search of all flips is only needed in the first iteration, while afterwards just a subset of possible flips should be considered which can be built either in a deterministic or probabilistic way.

Due to this lack of backpropagation, the flips are evaluated by their influence on the prediction when executed. However, when looking at a false positive, we can only correct this error by making the overall hypothesis of the network more specific. In order to achieve a generalization of the hypothesis, only flips from *false* to true in conjunctive layers or flips from true to false in disjunctive layers have to be taken into account. In this way, all flips are split into “generalization-flips” and “specialization-flips” of which only one group has to be considered at the same time. This improvement as well as the above-mentioned selection of a subset of flips might also allow us to perform two or more flips at the same time so that a better result than with the greedy approach can be achieved.

An even more promising approach starts one step earlier in the initialization phase of the network. Instead of specifying the structure of the network and finding optimal initialization parameters $\bar{l}$ and *p* for it, a small part of the data could be used to create a rough draft version of the network. The Quine-McCluskey algorithm (McCluskey, 1956), RIPPER and the ESPRESSO-algorithm (Brayton et al., 1984) are suitable methods to generate shallow networks, whereas decision trees and decision graphs can be used to generate deep networks since the contained rules already share some conditions and, moreover, similar subtrees can be merged.

All these approaches share some significant advantages over the network approach we developed so far. First of all, the decision which class value will be treated as positive or negative does not have to be made manually any longer. Second, they automatically deliver a suitable initialization of the network, which otherwise would have to be improved by similar approaches like used in neural networks (e.g., Ramos et al., 2017) to achieve a robust performance. Third, the general structure of the network is not limited to a fixed size and depth where each node is strictly assigned to a specific layer. Instead of generating nodes that become useless after a few flips have been processed and that should be removed, we can thereby start with a small structure which can be adapted purposefully by copying and mutating good nodes and pruning bad ones. However, it remains unclear whether these changes still lead to improvements in performance or if the network in the given structure is already optimal.

## Data Availability

The datasets presented in this study can be found in this online repository: https://github.com/f-beck/scikit-learn-rule-network.

## Footnotes

1 We use a Prolog-like notation for rules, where the consequent (the head of the rule) is written on the left and the antecedent (the body) is written on the right. For example, the first rule reads as: If x1, x2, x3 and x4 are all true and x5 is false then parity holds

2
https://github.com/f-beck/scikit-learn-rule-network

3 We used the python library SymPy (https://www.sympy.org/en/index.html) to compute the corresponding DNF representation

4 We used the implementations available from https://pypi.org/project/wittgenstein/and https://scikit-learn.org/stable/modules/generated/sklearn.tree.DecisionTreeClassifier.html.
